# Development of orally disintegrating tablets containing solid dispersion of a poorly soluble drug for enhanced dissolution: *In-vitro* optimization/*in-vivo* evaluation

**DOI:** 10.1371/journal.pone.0244646

**Published:** 2020-12-31

**Authors:** Shahinaze A. Fouad, Fady A. Malaak, Mohamed A. El-Nabarawi, Khalid Abu Zeid

**Affiliations:** 1 Department of Pharmaceutics, Faculty of Pharmacy, Ahram Canadian University, 6^th^ of October City, Giza, Egypt; 2 Department of Pharmaceutics and Industrial Pharmacy, Faculty of Pharmacy, Cairo University, Cairo, Egypt; Bahauddin Zakariya University, PAKISTAN

## Abstract

Diacerein (DCN), a potent anti-inflammatory API used to treat osteoarthritis yet, it suffers from poor water solubility which affects its oral absorption. Unabsorbed colonic DCN is converted into rhein, which is responsible for laxation as a main side effect of DCN treatment. Therefore, in this study orally disintegrating tablets (ODTs) loaded with optimized DCN solid dispersion system were prepared using different co-processed excipients (Prosolv^®^ ODT, Pharmaburst^®^ 500 and F-melt^®^), aiming to achieve improved solubility, rapid absorption and consequently limited amount of rhein reaching the colon. Prepared ODTs were evaluated for physical characteristics, *in*-*vitro* drug release, disintegration and wetting times. Dissolution parameters; dissolution efficiency percent at 10 (DE _(10 min)_%) and 30 (DE _(30 min)_%) min and mean dissolution time (MDT) were determined. The optimized ODT showed 1.50 and 1.12 fold increase in DE _(10 min)_% and DE _(30 min)_%, respectively and 2 fold decrease in MDT, compared to Diacerein^®^ capsules. *In*-*vivo* anti-inflammatory effect of optimized ODT, using rat paw edema revealed significant increase in edema inhibition (p < 0.0465) and promoted onset of action compared to Diacerein^®^ capsules at 0.5 hr. It could be concluded that optimized ODT could be promising for enhanced dissolution and rapid absorption of DCN from the oral cavity.

## 1. Introduction

Osteoarthritis (OA) is the most commonly known type of arthritis affecting middle aged and elderly populations worldwide. It is the predominant cause of joint pain and one of the most leading reasons for disability; as it affects joints of the hands, feet, knees, hips and spine [1]. It is also known as “degenerative joint disease” which is characterized by the break-down of cartilage in the affected joints. OA is unfortunately progressive and worsens over time, often resulting in chronic joint pain and stiffness which can become severe enough to make daily tasks difficult [2]. It is treated clinically via analgesia including both topical and oral non-steroidal anti-inflammatory drugs (NSAIDs), cyclo-oxygenase-II (COX-II) inhibitors [3] as well as acetaminophen, as first line treating agents. The most effective class of drugs is NSAIDs however, their continuous administration is associated with gastric irritation and cardiovascular diseases [4]. In addition, the aforementioned agents are responsible for reducing pain associated with OA but they impose potential toxicities and side effects which limit their use among treated populations. Being the first line treating agents for OA, they proved effectiveness in symptomatic pain relief yet, they are not considered to be disease modifying [5]. Therefore, developing alternatives to these treatments is increasing in demand.

Diacerein (DCN) is an anti-inflammatory drug, with analgesic effect that is widely used as a symptomatic treatment for OA [6]. Since inflammation is associated with disease initiation and progression, DCN was found to be a successful alternative to other problematic treatments. According to the European Society for Clinical and Economic Aspects of Osteoporosis and Osteoarthritis (ESCEO), it was confirmed that efficacy of DCN is comparable to that of NSAIDs and is better than that of acetaminophen in treating OA [7]. DCN exhibits both anti-inflammatory and chondro-protective activities. It works on two levels; the first one is inhibition of Interleukin-1β (IL-1β), which is the main cytokine involved in inflammation and destruction of cartilage in joints. The second level is activating the synthesis of proteoglycans and hyaluronic acid which are the main components of cartilages [8]. Thus, it possesses anti-catabolic and pro-anabolic properties by exerting a protective action on articular tissues, namely cartilages and synovial membranes [7]. The fact that DCN has no effect on prostaglandins synthesis, eliminates the severe gastrointestinal side effects coexisting with NSAIDs administration [9].

Although DCN is well known for its beneficial capacities in treating OA, it belongs to the Biopharmaceutics Classification System, class II (BCS II) therefore, it suffers from poor aqueous solubility (3.197 mg/mL) [10], which results in its limited dissolution and absorption [9], as well as its reduced oral bioavailability (35–56%) [11, 12]. Previous formulation strategies were performed to improve DCN solubility, dissolution properties and consequently its bioavailability. For instance, surfactant based solid dispersions of DCN were formulated via conventional melting method [11]. Also, inclusion complexes of DCN were prepared using β-cyclodextrin and hydroxypropyl β-cyclodextrin by kneading method [13]. DCN loaded solid lipid nanoparticles were developed via modified high shear homogenization and ultra-sonication [14]. In addition, niosomes as well as, mixed niosomes were assigned for entrapping DCN as a model for poorly water soluble drug molecules, using different surfactant/cholesterol ratios [15]. Within the same context, DCN nano-suspensions were developed via sono-precipitation followed by chitosan coating [16].

As a matter of fact, diarrhea is the main side effect associated with DCN [17]. This is explained by the fact that un-absorbed DCN in the gastrointestinal tract (GIT) is completely converted into rhein (causing laxation) before reaching the systemic circulation [14]. Hence, deficient and incomplete absorption of DCN makes rhein sufficiently available to the colon resulting in increased laxation [18]. In order to restrain rhein amount reaching the colon, orally disintegrating tablets (ODTs) containing optimized DCN solid dispersion (DCN-SD) were designed and evaluated in the current study, using commercially available co-processed excipients. Literature lacks any data about loading DCN-SD into ODTs using co-processed excipients for enhanced dissolution and rapid oral absorption. In a previous study, we investigated how solid dispersion systems of DCN, prepared using hydrophilic polymers, succeeded in enhancing DCN dissolution properties. Previously prepared optimized DCN-SD system exhibited the maximum enhanced dissolution parameters and improved bioavailability compared to plain DCN (research article is under review in PLOS ONE Journal having reference number PONE-D-20-25781R1). Thus, the optimized DCN-SD was chosen as a foundation for preparation of DCN-ODTs in the current study.

ODTs are oral solid dosage forms that disintegrate rapidly in the oral cavity releasing the drug. They contain superdisintegrants that helps in dissolving the ODT without water intake within three seconds (s) to three minutes (min) [19]. This makes ODTs advantageous to many populations of patients including geriatrics and also increases their compliance [20]. Because the oral cavity is rich in blood supply, drugs are directly delivered to the systemic circulation resulting in rapid absorption, enhanced bioavailability and reduced side effects.

In the current study, formulations are prepared by the direct compression technique. It is the most simple and economic method for conventional tablet press [19]. However, achieving robust disintegration and enhanced tablet porosity together with sufficient mechanical strength is a critical challenge. Among the various strategies employed to produce adequately hard ODTs without impairing disintegration time is “co-processing of excipients”. Co-processing methods include mixing, co-grinding and spray drying. These processes produce ODTs incorporable excipients, having enhanced mechanical properties with preserved disintegration times. Mannitol-based co-processed excipients are one of the most common ODTs excipients that are commercially available in the pharmaceutical market and that are ready to use. Although these excipients may be very similar in composition yet, different fabrication methods, as well as minor changes in the characteristics of their components make them react otherwise after ODT formation [21]. Examples of active pharmaceutical ingredients (APIs) that were formulated as ODTs using co-processed excipients include sertraline hydrochloride [22], chlorpeniramine maleate [23] and chlorzoxazone [24].

The aim of this study is to formulate, evaluate and optimize Diacerein orally disintegrating tablets (DCN-ODTs) to provide robust drug release, rapid absorption and enhanced anti-inflammatory effect by comparing the optimized DCN-ODT with the conventional commercially available oral Diacerein^®^ capsules in rats, using the rat paw edema method.

## 2. Materials and methods

### 2.1. Materials

DCN was kindly supplied by EVA Pharm Egypt. Pharmaburst^®^ 500 was received from SPI pharma, Wilmington, DE, USA. Prosolv^®^ ODT was a gift from JRS pharma GmbH & Co. KG, Rosenberg, Germany. F-melt^®^ type C was a kind gift from Fuji Chemical Industry Ltd., Toyama-Pref, Japan. Disodium hydrogen orthophosphate, potassium dihydrogen orthophosphate were supplied from El-Nasr Company for pharmaceuticals, Cairo, Egypt. Distilled water was used throughout the study. All other chemicals and solvents were reagent grade and used as received.

### 2.2. Preparation of Diacerein Orally Disintegrating Tablets (DCN-ODTs)

Based on our previous research work (research article under review in PLOS ONE Journal having reference number PONE-D-20-25781R1), an optimized DCN-SD was selected as a base for preparing DCN-ODTs. It contained DCN and polyethylene glycol (PEG) 8000 in a ratio 1:4 w/w. DCN-ODTs were prepared adopting the direct compression method using a single punch tablet machine under a constant pressure using concave-faced 9 mm punch and die set. Three types of ready-made co-processed excipients (Prosolv^®^ ODT, Pharmaburst^®^ 500 and F-melt^®^) were added to the previously prepared optimized DCN-SD in different amounts, to give a final weight of 500 mg tablet. The powder blend of each tablet was fed manually into the die and compressed into tablets.

### 2.3. Formulation optimization

I-Optimal mixture experimental design was employed to evaluate the effect of formulation variables on the properties of the prepared ODTs using Design Expert^®^ software (Version 10.0.3, Stat-Ease Inc. Minneapolis, MN, USA). In this design, three independent variables were studied which were concentrations of each of Prosolv^®^ ODT (X_1_), Pharmaburst^®^ 500 (X_2_) and F-melt^®^ (X_3_). The selected dependent variables were the dissolution efficiency % at 10 min (Y_1_: DE _(10 min)_ %), the dissolution efficiency % at 30 min (Y_2_: DE _(30 min)_ %), the mean dissolution time (Y_3_: MDT), the disintegration time (Y_4_: DT) and the wetting time (Y_5_: WT). The Composition of DCN-ODTs is listed in Table (2) in S1 File.

### 2.4. Evaluation of the prepared ODTs

#### 2.4.1. Physical characterization

The prepared ODTs were evaluated according to the compendial specifications [25]. Physical characterization was performed by carrying out tests for friability, thickness, hardness, content uniformity and weight uniformity. All tests were done in triplicate (n = 3 ± S.D.).

#### 2.4.2. In-vitro Disintegration Time (DT) test

Disintegration times of the prepared ODTs were determined. Each individual tablet was dropped into a beaker containing 5 mL of simulated saliva fluid (SSF) pH 6.8 at 37 ± 0.5°C, and the time required for complete tablet disintegration was observed visually and recorded using a stop watch [24]. All experiments were performed in triplicate (n = 3 ± S.D.) and all results were expressed as mean ± S.D.

#### 2.4.3. Wetting Time (WT) test

A piece of tissue paper having dimensions 10.75 × 12 mm, was folded twice and immersed in a petri dish (6.5 cm in diameter), containing 6 mL distilled water colored with methylene blue dye (2% w/v). A tablet was carefully placed on the surface of the tissue paper and the time required for the dye solution to reach the upper surface of the tablet was accurately determined as the wetting time [26]. The colored dye was employed to enable appropriate end point observance. This test is considered a simple simulation to the physiologically wet surface of the tongue however, the influence of mechanical stress exerted by the human tongue is disregarded [27]. All determinations were done in triplicate (n = 3 ± S.D.) and all results were expressed as mean ± S.D.

#### 2.4.4. *In-vitro* dissolution study

Dissolution profiles of DCN from the prepared ODTs compared to plain drug and Diacerein^®^ capsules were determined using U.S.P dissolution tester (Apparatus II) at 37°C ± 0.5 and 50 revolutions per minute (r.p.m). The used dissolution medium was 900 mL SSF (pH 6.8) [24]. At specified time intervals (2, 4, 6, 10, 15, 20, 25, 30, 45 and 60 min), 5 mL samples were withdrawn and replaced with an equal volume of fresh dissolution medium to maintain a constant total volume. The collected samples were filtered through 0.45 μm membrane filter, suitably diluted with SSF (pH 6.8) and analyzed spectrophotometrically at DCN λmax. *In*-*vitro* dissolution tests were carried out in triplicate (n = 3 ± S.D.). Also, similarity factor (*f*2), a model-independent mathematical approach, was calculated to compare the dissolution profiles of pure drug with the prepared formulations, as well as the commercial product, using the following equation [28]:

$$f_{2}=50\log\{{\left[1+\frac{1}{n}\sum_{t=1}^{n}{\left(R_{t}-T_{t}\right)}^{2}\right]}^{0.5}x100\}$$ Eq (1)

*f*2 values less than 50 indicates significant difference between dissolution profiles.

#### 2.4.5. Determination of Dissolution Efficiency (DE) and Mean Dissolution Time (MDT)

Dissolution of sparingly soluble drugs is a prerequisite for their gastrointestinal absorption therefore, factors influencing their dissolution enhancement should be thoroughly evaluated. Two-point dissolution specification test is required for drugs belonging to BCS II, in order to assure 85% dissolution [29]. Hence, dissolution efficiency percent (DE %) at 10 and 30 min was calculated for all formulations, as well as plain drug and the commercial product, using the following equation [30]:

$$Dissolution\;efficiency\;(\%)=\frac{\int_{t1}^{t2}ydt}{y_{100}(t_{2}-t_{1})}\times 100$$ Eq (2)

Similarly, this term has been explored by many researchers to observe the extent of drug dissolution from different formulations compared to plain drug [31, 32].

In addition, mean dissolution time (MDT) was employed to determine the rate of drug release from the prepared formulations. It was calculated using the following formula [33]:

$$MDT=\frac{\sum_{j=1}^{n}t_{j}^{*}\Delta M_{j}}{\sum_{j=1}^{n}\Delta M_{j}}$$ Eq (3)

#### 2.4.6. Statistical analysis

Analysis of variance (ANOVA) was carried out to estimate the significance of model and term. Probability, p-values, (p < 0.05) denoted significance. Desirability was calculated for selection of the optimized formulation which was prepared and subjected to further investigations.

#### 2.4.7. *In-vivo* study of anti-inflammatory effect of DCN-ODTs

The enhanced solubility and anti-inflammatory activity of DCN was tested *in*-*vivo* using carrageenan-induced rat paw edema method as an acute inflammatory inducing model [34]. In this experiment, commercially available Diacerein^®^ capsules were used as a reference standard.

In this study, eighteen adult male albino wistar rats, weighing 200–250 g were employed. They were purchased from Helwan’s Farm for experimental animals (Cairo, Egypt). The animals were adapted to the laboratory’s environment for one week where, they were housed under controlled environment at 25 ± 1°C with a 12 hr light/dark cycle. All animals had free access to standard rodent pellet food and water [35]. Rats were divided into three groups, each group is composed of six rats. The first group was the positive control group where each rat was injected with 0.1 mL of 1% carrageenan suspension in 0.9% saline solution into the sub plantar region of the left hind paw [36] of each animal [37]. Optimized DCN-ODTs were assigned to the second group and Diacerein^®^ capsules were assigned to the third group.

At the beginning of the experiment, the optimized DCN-ODTs and the reference standard Diacerein^®^ were administered at a dose equivalent to 5 mg/kg p.o. [38]. After one hour of oral administration of the assigned doses, all groups were injected with 0.1 mL of 1% carrageenan suspension in 0.9% saline solution into the sub plantar region of the left hind paw of each animal. The paw volume was measured using the Plethysmometer (Ugo Basile 37140, Italy) for the positive control group (Vi), and the groups after treatment (Vt), at different time intervals 0.5, 1, 2, 3 and 4 hrs. In order to calculate the percent of edema volume, the following equation was used [35]:

$$\%\;edema\;volume=\left(\frac{Vt-Vi}{Vi}\right)\times 100$$ Eq (4)

Data were expressed as mean values of treated animals ± SEM. Data were statistically analyzed using one-way ANOVA at 5% significance level followed by Tukey Kramer’s test using GraphPad Prism 6 software (GraphPad Inc., USA). Experimental procedures including the *in*-*vivo* study protocol were reviewed and approved by the Research Ethics Committee (REC) for experimental and clinical studies at Faculty of Pharmacy, Cairo University, Cairo, Egypt (serial number of protocol is PI (1782)).

## 3. Results and discussion

### 3.1. Physical characterization

All formulations resulted in successful elegant drug-loaded ODTs. All physical characterization tests were within the Pharmacopoeia limit. All tablets showed friability < 1% (S1 Table). Values of friability less than 1% indicates good mechanical strength and ability to tolerate physical handling conditions [39]. Hardness values for the prepared formulations ranged from 4.9 ± 0.2 Kg to 5.8 ± 0.2 Kg. Since the force of compression was the same for all formulations, the change in hardness values could be due to the concentration and the type of co-processed excipients used. None of the prepared ODTs showed hardness below 3kg. According to literature, hardness of ODTs is preferable to range between 2–8 Kg [24], where it provides sufficient mechanical strength and preserved porosity for quick disintegration and wetting times. The average thickness of tablets ranged from 3.23 ± 0.04 mm to 3.27 ± 0.06 mm. The mean percentage of DCN content within the prepared tablets ranged from 96.19 ± 2.34% to 101.23 ± 0.78% which conforms with the pharmacopoeia limit (85–115%) [40]. The prepared tablets were within the accepted weight variation range according to the European Pharmacopoeia (S1 Table) [25].

### 3.2. Formulation optimization of DCN-ODTs using I-Optimal mixture experimental design

Optimization of formulations aims to detect levels of formulation variables from which an optimized pharmaceutical product can be produced. This is because identifying variables that can affect the properties of a new formulation is necessarily required [41]. Moreover, experiments with mixture of variables require good prediction properties from the estimated model in order to reach the optimized formulation [42]. Hence, an I-Optimal mixture design was recruited in this study as it minimizes the average prediction of variance throughout the experimental region [43].

In this study, three different co-processed excipients were selected for direct compression of the optimized DCN-SDs into ODTs. Generally, each co-processed excipient is composed of a mixture of two excipients or more aiming to increase the functionality of each sole excipient [44]. Therefore, they are considered sufficient for direct compression as they exclude the incorporation of multi-excipients within the formulation [45]. These excipients entail fillers, binders, superdisintegrants and sweeteners. Their existence in combined blends fulfills the special requirements needed for ODTs development including complete dissolution, rapid wetting and disintegration times, as well as pleasant mouth-feel and taste [46].

The effects of formulation variables on dissolution, as well as disintegration and wetting times behavior of DCN-ODTs were investigated. The studied independent variables were concentrations of Prosolv^®^ ODT (X_1_), Pharmaburst^®^ 500 (X_2_) and F-melt^®^ (X_3_) (Table 1). The studied responses (dependent variables) were the dissolution efficiency % at 10 min (Y_1_: DE _(10 min)_ %), the dissolution efficiency % at 30 min (Y_2_: DE _(30 min)_ %), the mean dissolution time (Y_3_: MDT), the disintegration time (Y_4_: DT) and the wetting time (Y_5_: WT). Responses of the prepared ODTs are listed in Table (2) in S1 File. Both dependent and independent variables were related using polynomial equation via statistical analysis using Design Expert^®^ software. ANOVA test was implemented for evaluating the significance of the model at 5% significance level where the model is considered significant when the p value ≤ 0.05. Contour plots were executed to point out the significant impact of independent variables on the measured responses, as well as to select the optimum levels of each variable.

**Table 1:** I-Optimal mixture design used to optimize DCN-ODTs.

Independent variables	Concentration range (0–250 mg)					
X_1_: Prosolv^®^ ODT concentration	0.00	62.50	83.30	125.00	―	250.00
X_2_: Pharmaburst^®^ 500 concentration	0.00	62.50	83.30	125.00	166.60	250.00
X_3_: F-melt^®^ concentration	0.00	62.50	83.30	125.00	―	250.00
Dependent variables	Constraints					
Y_1_: DE _(10 min)_ %	Maximize					
Y_2_: DE _(30 min)_ %	Maximize					
Y_3_: MDT (min)	Minimize					
Y_4_: DT (s)	Minimize					
Y_5_: WT (s)	Minimize					

**Table 2:** Experimental runs, formulation variables and measured responses of the I-Optimal mixture experimental design^a^^,^^b^.

Runs	Co-processed excipients			Responses				
	Prosolv^®^ ODT	Pharmaburst^®^ 500	F-melt^®^	Y_1_: DE _(10 min)_ %	Y_2_: DE _(30 min)_ %	Y_3_: MDT (min)	Y_4_: DT (s)	Y_5_: WT (s)
F 1	83.30	83.30	83.30	32.74	67.65	10.58	40 ± 3.50	150 ± 8.40
F 2	83.30	83.30	83.30	32.86	68.02	9.70	42 ± 4.10	180 ± 5.89
F 3	62.50	62.50	125.00	52.54	81.32	6.13	19.9 ± 3.50	42 ± 5.47
F 4	62.50	62.50	125.00	51.57	79.48	6.60	21 ± 6.50	45 ± 6.78
F 5	62.50	125.00	62.50	67.37	90.66	3.90	21.59 ± 2.40	39 ± 5.47
F 6	62.50	125.00	62.50	66.19	87.89	4.18	20.58 ± 5.80	38 ± 2.78
F 7	125.00	62.50	62.50	58.65	85.44	4.06	32 ± 9.70	132 ± 8.95
F 8	125.00	62.50	62.50	56.43	84.16	5.18	30 ± 10.23	127 ± 5.78
F 9	0.00	125.00	125.00	67.38	91.02	3.81	18.9 ± 5.80	38.4 ± 6.42
F 10	0.00	166.60	83.30	75.51	93.63	2.72	17.63 ± 6.80	35.2 ± 3.75
F 11	250.00	0.00	0.00	41.36	71.39	8.81	> 180	> 180
F 12	250.00	0.00	0.00	43.15	71.37	8.45	> 180	> 180
F 13	0.00	250.00	0.00	47.02	76.93	7.29	> 180	180 ± 11.49
F 14	0.00	250.00	0.00	47.69	76.90	7.32	> 180	174 ± 9.37
F 15	0.00	0.00	250.00	44.33	76.25	7.45	> 180	> 180
F 16	0.00	0.00	250.00	44.35	76.26	7.39	> 180	> 180

^a^Each formulation contained 250 mg solid dispersion of DCN (DCN:PEG 8000 with ratio 1:4 w/w).

^b^All weights are in mg/ODT.

### 3.3. *In*-*vitro* dissolution

**Fig 1:**
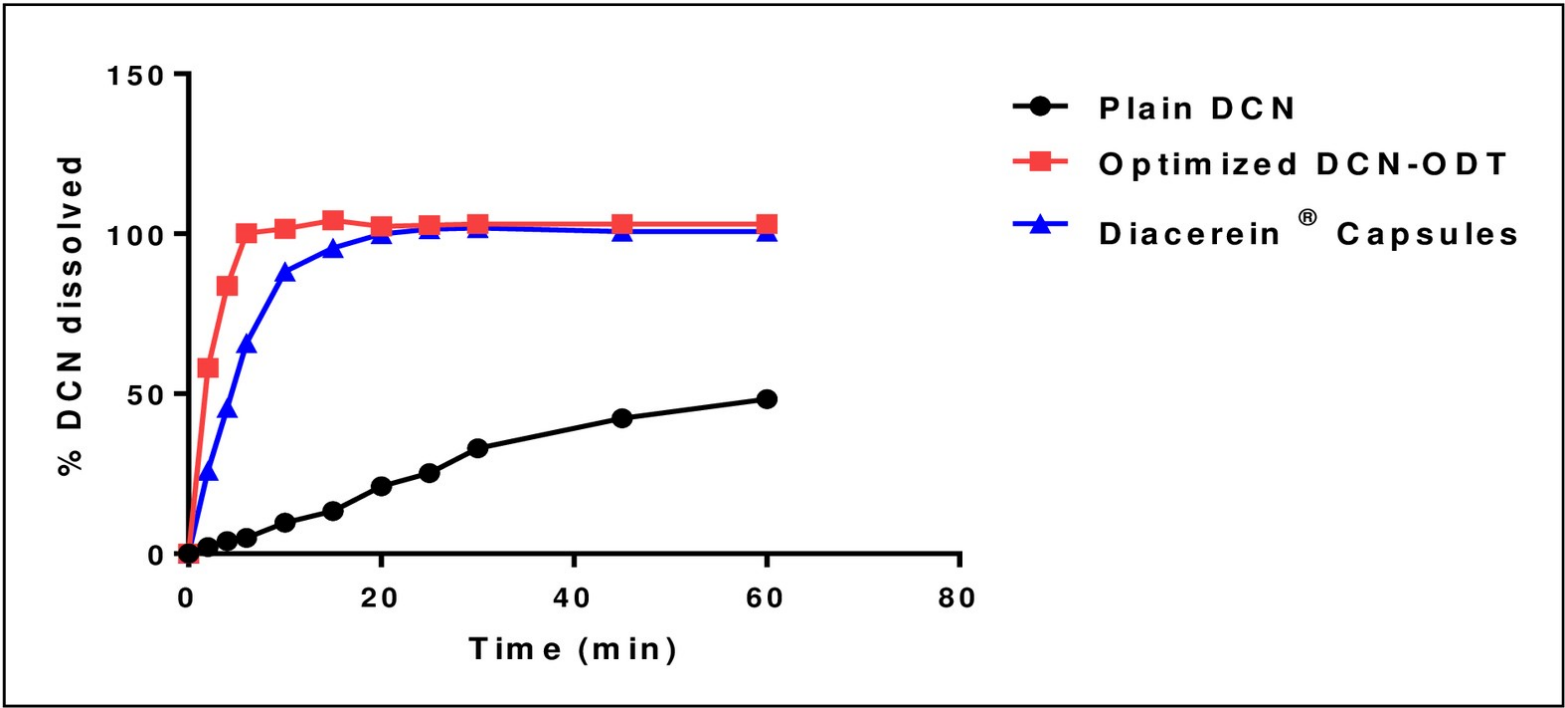
*In-vitro* mean dissolution profiles of DCN from optimized DCN-ODT (equivalent to 50 mg) compared to plain DCN and Diacerein^®^ capsules in phosphate buffer (pH 6.8).

“Fig 1” shows the enhanced drug dissolution of the optimized DCN-ODT compared to plain drug and the commercial product, Diacerein^®^ capsules. The crystalline drug powder showed a clearly slow and incomplete dissolution where, only 48.34 ± 2.01% was dissolved during the complete release duration (one hour). On the other hand, the optimized ODT showed complete and very rapid dissolution with a 10.5 fold increase in the percentage of drug dissolved compared to plain drug, after only ten min of the release duration. *f*2 calculated to compare dissolution profiles of plain DCN and the optimized ODT was found to be 6.67. Having a value less than 50, indicates significant difference between their dissolution profiles. Also, the optimized ODT showed a significantly higher dissolution rate than Diacerein^®^ capsules with *f*2 equals to 35.28. In addition, results showed that formulations (F1, F2, F5, F6, F9, F10, F11and F12) showed significantly higher dissolution profiles compared to the conventional product (*f*2 < 50). As previously mentioned, all DCN-ODTs were formulated using their optimized PEG 8000-based solid dispersion system. Hence, initial rapid drug dissolution was guaranteed within all formulations. This is because PEG 8000 prevented aggregation of drug particles and provided greater surface area for drug dissolution [47]. Inclusion of co-processed excipients within the prepared ODTs showed additional enhancement in DCN dissolution. This can be attributed to the hydrophilic components incorporated within the co-processed excipients. All excipients are mannitol-based. Also, Pharmaburst^®^ 500 contains additional sorbitol. These hydrophilic agents incite faster drug wetting, solubilization and enhanced drug release from ODTs. The imparted hydrophilicity allowed dissipation of drug particles upon their contact with the dissolution medium. Similar results were obtained by Brniak et al. in their study on prednisolone ODTs [48].

### 3.4. Statistical analysis

#### 3.4.1. Effect of formulation variables on DE _(10 min)_ %, DE _(30 min)_ % and MDT

**Fig 2:**
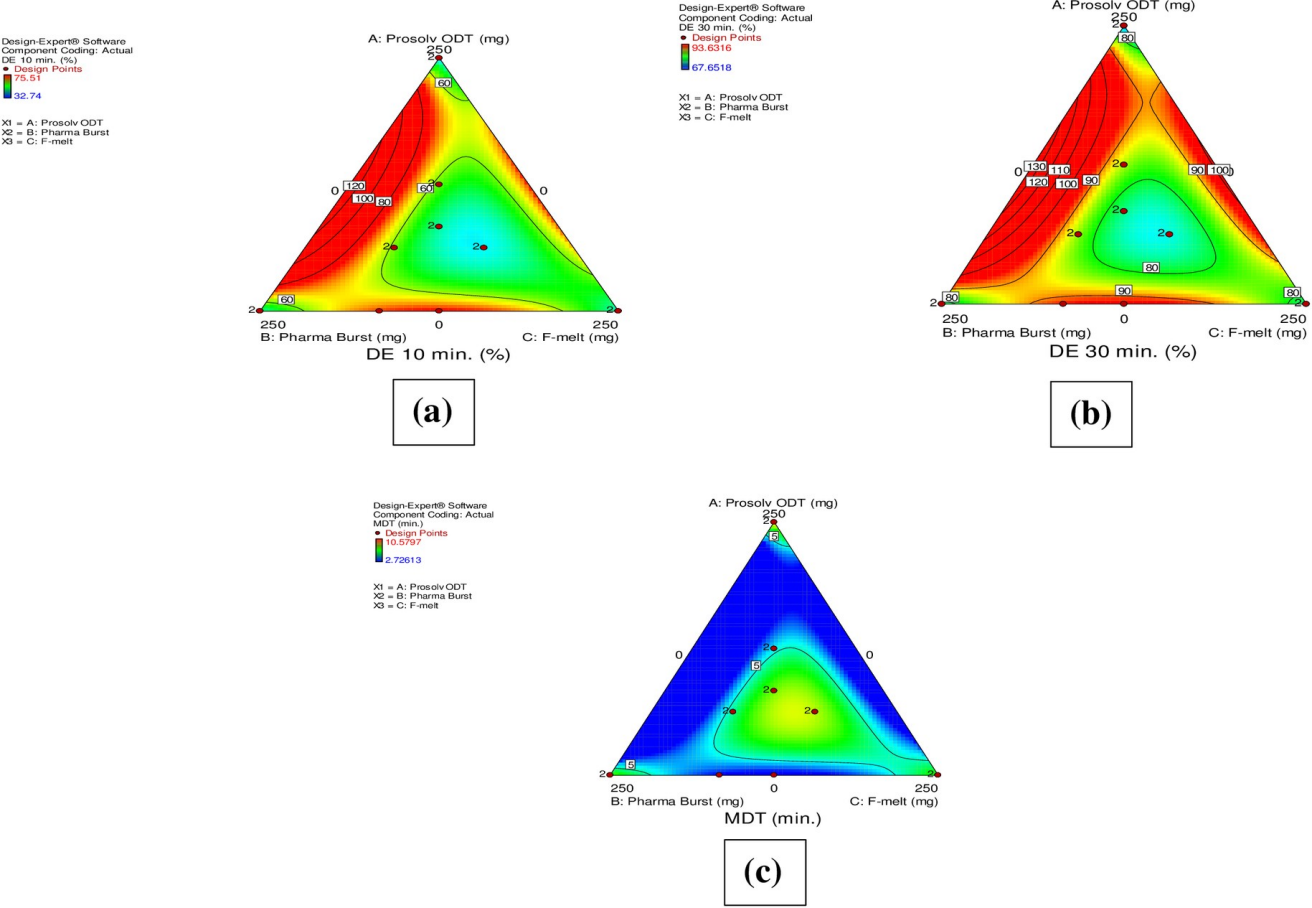
Contour plots of the effect of formulation variables on DE _(10 min)_ % (Y_1_) (a), DE _(30 min)_ % (Y_2_) (b) and MDT (Y_3_) (c) on DCN-ODTs.

Enhanced DCN dissolution is spontaneously reflected on dissolution efficiency percent of ODTs. Table 2 shows values of the dissolution efficiency percent ranging from 32.74% to 75.51% and 67.65% to 93.63% at 10 and 30 min, respectively. It also shows that MDT values of ODTs varied between 2.72 and 10.58 min. “Fig 2A, 2B and 2C” show the contour plot diagrams illustrating the effect of different concentrations of the co-processed excipients on DE _(10 min)_ % (Y_1_), DE _(30 min)_ % (Y_2_) and MDT (Y_3_), respectively. It can be concluded that the three independent variables had a significant effect on DE _(10 min)_ %, DE _(30 min)_ % and MDT of DCN-ODTs where, ANOVA indicated a p-value of < 0.0001 for all the independent variables. It is clearly known that values of coefficients X_1_, X_2_ and X_3_ are allied to the effect of independent variables on the measured responses. Synergistic effect is conjuncted with the positive sign of the coefficient and antagonistic effect with its negative sign [35]. The greater the coefficient, the more it becomes influential upon the response. Table 3 confirms the significant effect of X_1_, X_2_ and X_3_ on Y_1_ and Y_2_ through the positive value of each of these coefficients either alone or in their dual combinations. However, their triple existence showed a significant synergistic effect upon them.

**Table 3:** Statistical parameters for the measured responses.

Response	Model p-value	R^2^	Adjusted R^2^	Predicted R^2^	PRESS	Polynomial equations for the responses
Y_1_	< 0.0001	0.9965	0.9942	0.9900	15.28	Y_1_ = +42.76X_1_ +48.28X_2_ +44.80X_3_ +341.94X_1_X_2_ +127.86X_1_X_3_ +111.51X_2_X_3_−17140.9X_1_X_2_X_3_
Y_2_	< 0.0001	0.9996	0.9993	0.9988	33.69	Y_2_ = +71.04X_1_ +77.36X_2_ +76.55X_3_ +249.90X_1_X_2_ +111.15X_1_X_3_ +70.36X_2_X_3_−1239.13X_1_X_2_X_3_
Y_3_	< 0.0001	0.9976	0.9960	0.9920	0.46	Y_3_ = +8.85X_1_ +7.11X_2_ +7.23X_3_−79.09X_1_X_2_−42.37X_1_X_3_−19.53X_2_X_3_ +416.57X_1_X_2_X_3_
Y_4_	< 0.0001	0.9971	0.9952	0.9893	28736.54	Y_4_ = +1068.65X_1_ +567.76X_2_ +855.15X_3_−3692.29X_1_X_2_−4852.03X_1_X_3_−2841.98X_2_X_3_ +12371.61X_1_X_2_X_3_
Y_5_	< 0.0001	0.9989	0.9983	0.9971	397.86	Y_5_ = +309.58X_1_ +174.90X_2_ +263.64X_3_−189.60X_1_X_2_−464.56X_1_X_3_−782.24X_2_X_3_

Also, positive effect of each of X_1_, X_2_ and X_3_ on Y_3_ is observed by their presence alone and in triple combination only. Results showed that formulations with high dissolution efficiency percent attained lower MDT values and vice versa. F1 (composed of equal concentrations of the three co-processed excipients) exhibited the least DE _(10 min)_ % (32.74%), DE _(30 min)_ % (67.65%) and the longest MDT (10.58 min) among the prepared formulations. These results cohere well with the polynomial equations for the three responses (Table 3). It is clear that ternary mixture of co-processed excipients produced a synergistic effect upon responses under study. On the other hand, F10 attained the highest DE _(10 min)_ % (75.51%), DE _(30 min)_ % (93.63%) and the least MDT (2.72 min). F10 contains a high concentration of Pharmaburst^®^ 500 which contains crospovidone; a superdisintegrant. This enables rapid absorption of fluids by capillarity (wicking) into the tablet, accompanied by swelling, resulting in rapid and enhanced dissolution. Similar results were obtained by Zayed et al. in their study on sildenafil citrate sublingual tablets [49].

#### 3.4.2. Effect of formulation variables on *in*-*vitro* Disintegration Times (DT) and Wetting Times (WT) of DCN-ODTs

**Fig 3:**
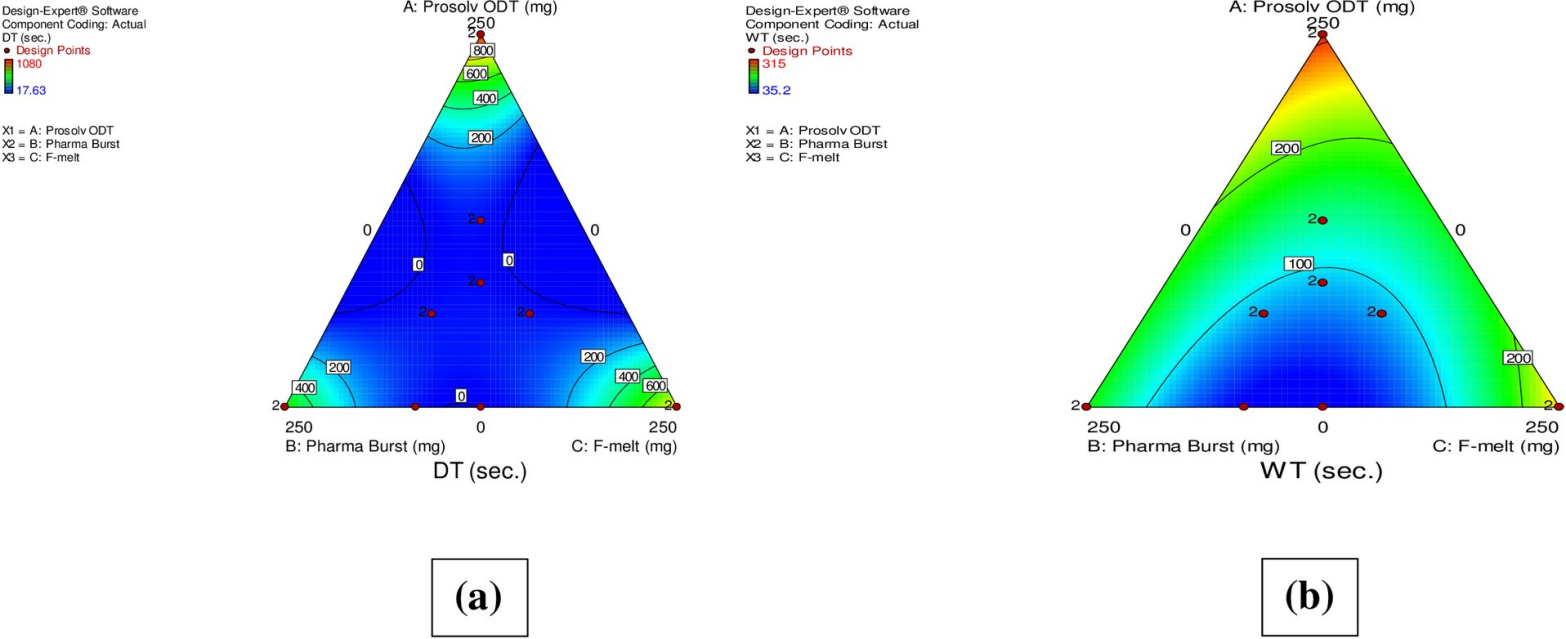
Contour plots of the effect of formulation variables on DT (Y_4_) (a) and WT (Y_5_) (b) on DCN-ODTs.

DT strongly pertains to WT of the prepared ODTs where lowered WT indicates faster DT. Generally, disintegration of ODTs prepared by direct compression depends on the individual and combined effects of the used co-processed excipients [50]. It is also greatly affected by the included disintegrants and hydrophilic components within the formulation [24]. The European pharmacopeia established a time limit of 180 s for disintegration of ODTs [25]. Table 2 displays the DT (s) and WT (s) of the prepared formulations. “Fig 3A and 3B” show the contour plots demonstrating the effect of formulation variables on DT and WT, respectively. ANOVA results revealed that the three independent variables had a significant effect on both DT and WT of ODTs (p < 0.0001). In our study, formulations containing mixture of two or three co-processed excipients showed DT within the pharmacopeia limit [25]. On the other hand, formulations (F11-F16) composed of only one type (= 250 mg) of the used co-processed excipients, exceeded the acceptable time for disintegration (DT > 180 s). A possible justification is that all co-processed excipients under study are mannitol-based. Therefore, their presence alone with a maximized concentration (as shown in Table 1), spontaneously increases mannitol content. This in return leads to increased binding capacity and hydrogen bond formation between mannitol and DCN, which could be the suggested reason for DT exceeding the pharmacopeia limit within these formulations. Also, formulations containing only Prosolv^®^ ODT (F11-F12) and only F-melt^®^ (F15-F16) showed highly exceeded DT compared to formulations containing Pharmaburst^®^ 500 (F13-F14) only. This is because these excipients contain microcrystalline cellulose (MCC) but Pharmaburst^®^ 500 does not. As a result, these formulations acquire more hard core matrix that detains DT. Jacob et al. observed similar results. They stated that microcrystalline cellulose (MCC) and mannitol exhibit non-wetting properties and central rigid core leading to delayed disintegration [51].

By comparing the effect of co-processed excipients on these two variables, it was clear that Prosolv^®^ ODT had the highest positive effect on DT and WT of the prepared ODTs. Increasing Prosolv^®^ ODT concentration resulted in greater prolongation of both DT and WT of the prepared ODTs. This could be attributed to the complex composition of Prosolv^®^ ODT containing MCC, mannitol, crospovidone, colloidal silicon dioxide and fructose. This composite matrix of Prosolv^®^ ODT enhanced compactability in favor of disintegration [27]. Lahdenpaa et al. similarly reported that the compactable nature of Prosolv^®^ ODT exerts a remarkable increase in tablets’ DT [52]. Also, similar results were obtained by Tayel et al. in their study on Sumatriptan Succinate sublingual tablets [21]. Wetting properties of ODTs containing Prosolv^®^ ODT were significantly decreased because water sorption was strongly lowered by the effect of silicified MMC contained within Prosolv^®^ ODT [52]. Also, Sunada et al. confirmed that high MCC content resulted in lower porosity (due to lower water uptake) and detained WT [26]. Another reason for moisture sorption drop-off in formulations containing high concentrations of Prosolv^®^ ODT is attributed to the inclusion of mannitol within the Prosolv^®^ ODT matrix. Mannitol, having a non-hygroscopic property [53], may cover the silicified MCC fibres leading to a further decrease in ODTs’ porosity resulting in detained WT.

F-melt^®^ based formulations showed shorter DT and WT compared to Prosolv^®^ ODT based ones. This is because of the included dibasic calcium phosphate (insoluble inorganic salt) together with crospovidone (superdisintegrant) which resulted in shortened DT and WT with advantageous improved mechanical resistance of ODTs [54]. Similar results were also reported by Dobetti in his study on fast melting tablets where, water insoluble inorganic excipients provided enhanced disintegration properties than the frequently used water soluble salts and sugars [50].

Increasing Pharmaburst^®^ 500 concentrations showed notable decrease in both DT and WT compared to Prosolv^®^ ODT and F-melt^®^. This may be ascribed to the included sorbitol in addition to mannitol within Pharmaburst^®^ 500 compared to the presence of mannitol only in Prosolv^®^ ODT and F-melt^®^. The presence of an equatorial hydroxyl group on the C-2 atom in Sorbitol led to increased hydration and enhanced the wetting capacity than that in case of the axial hydroxyl group in mannitol. This is because equatorial hydroxyl groups are capable of forming two hydrogen bonds compared to only one hydrogen bond formation in case of axial hydroxyl groups [21]. The influence of axial and equatorial distributions of hydroxyl groups could possibly be interpreted by crystal arrangement of molecules. Similar results were obtained by Tayel et al in their study on Sumatriptan Succinate sublingual tablets [21]. In addition, F-melt^®^ has lower specific surface area compared to Pharmaburst^®^ 500 [24] which results in lower DT within F-melt^®^ based formulations. Results revealed that the shortest DT (17.63 ± 6.80) and WT (35.2 ± 3.75) were observed by F10 which contained high concentration of Pharmaburst^®^ 500 and does not contain Prosolv^®^ ODT. These results were in accordance with the dissolution efficiency and MDT results.

### 3.5. Preparation of optimized DCN-ODT

**Fig 4:**
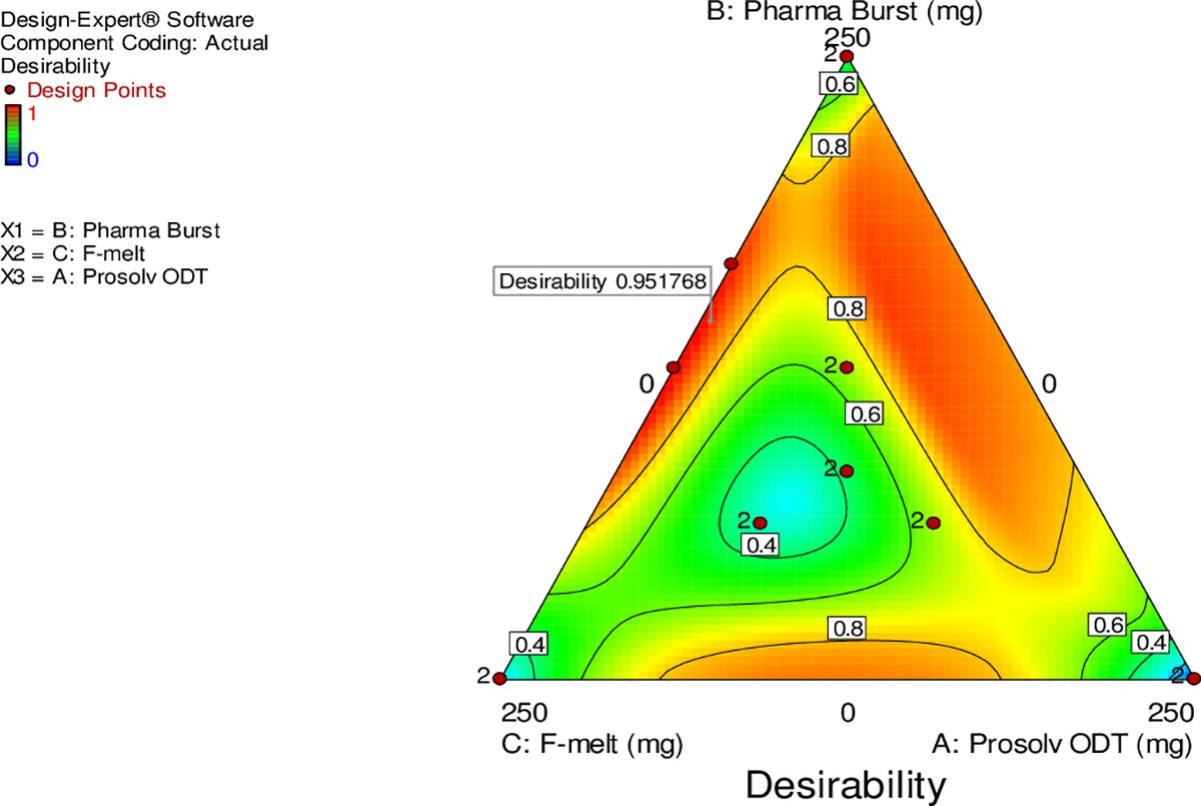
Overlay plot for the effect of formulation variables on the measured responses.

In order to produce a formulation of the required properties, some of the responses have to be maximized, while others have to be minimized. The selected optimized formulation was prepared based on desirability calculations in order to accomplish maximized DE _(10 min)_ % and DE _(30 min)_ % as well as minimized MDT, DT and WT. The desirability function recruits all the responses into a single variable in order to expect the optimum levels of factors under investigation [41]. These factors were combined to obtain an overall optimum region meeting the criteria of the measured responses (“Fig 4”). The optimized ODT was then prepared and evaluated in triplicate to ensure coherence of the measured responses given by the program software. The predicted optimized DCN-ODT is composed of 250 mg optimized DCN-SDs (composed of DCN:PEG 8000 in a ratio of 1:4 w/w), 134.6 mg Pharmaburst^®^ 500 and 115.4 mg F-melt^®^. Results showed high coherence between the observed and the predicted values of the optimized DCN-ODT (Table 4). The optimized formulation showed 16.5 and 6.2 fold increase in DE _(10 min)_ %, and DE _(30 min)_ %, respectively compared to plain DCN. It also showed 1.5 and 1.12 fold increase in DE _(10 min)_ %, and DE _(30 min)_ %, respectively compared to Diacerein^®^ capsules. The optimized formulation showed a significant decrease in MDT, 9.24 and 2 fold compared to plain DCN powder and Diacerein^®^ capsules, respectively (p < 0.0001). It was also evaluated for physical characterization including; weight variation (499.22 ± 3.68 mg), thickness (3.24 ± 0.05 mm), friability (0.953%), hardness (4.8 ± 0.6 Kg) and drug content (97.20 ± 2.04%). All results were within the pharmacopeia limit.

**Table 4:** Predicted and observed responses for the optimized DCN-ODT.

Responses	Predicted	Observed	Residual*
Y_1_: DE _(10 min)_ %	74.39	76.02	-1.63
Y_2_: DE _(30 min)_ %	94.48	93.92	0.554
Y_3_: MDT (min)	2.31	2.69	-0.381
Y_4_: DT (s)	5.88	14.32	-8.44
Y_5_: WT (s)	21.45	29.58	-8.13

*Residual = Predicted values–Observed values.

### 3.6. *In-vivo* study of the anti-inflammatory effect of DCN

The anti-inflammatory activity of DCN was postulated to measure the enhanced absorption of DCN, as well as its onset of action, compared to the commercially available Diacerein^®^ capsules (used as a reference standard). The model for inflammation used in this study is the “rat hind paw edema” method. In this method, carrageenan solution was selected as a phlogistic agent; an irritant inducing inflammation [55]. Results showed that it induced remarkable edema in the injected rats’ groups. The anti-inflammatory activity of a single oral dose of the optimized DCN-ODT (equivalent to 5 mg/Kg) was tested compared to Diacerein^®^ capsules, having the same dose at different time intervals.

**Fig 5:**
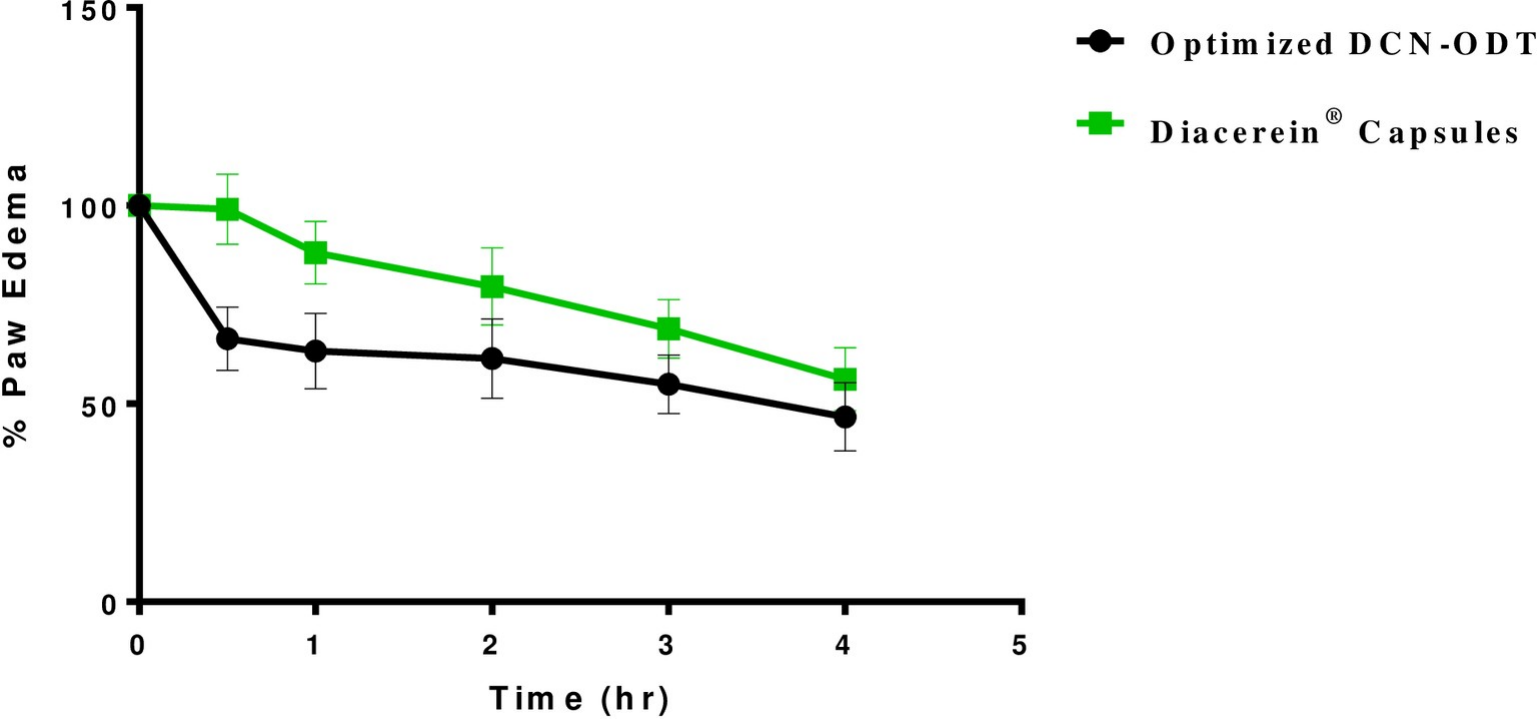
Anti-inflammatory activity of optimized DCN-ODT compared to Diacerein^®^ capsules using the rat paw edema model.

As shown in “Fig 5”, the highest inhibition of edema was denoted at 0.5 hr after dose administration. This result was observed with the optimized formulation. Optimized DCN-ODT exhibited a 1.5 fold increase in edema inhibition at 0.5 hr compared to Diacerein^®^ capsules (p = 0.0465). Edema inhibition was extended through the whole duration of the experiment. However, it turned to be nearly the same for the tested formulations (p = 0.135) at 4 hrs.

**Fig 6:**
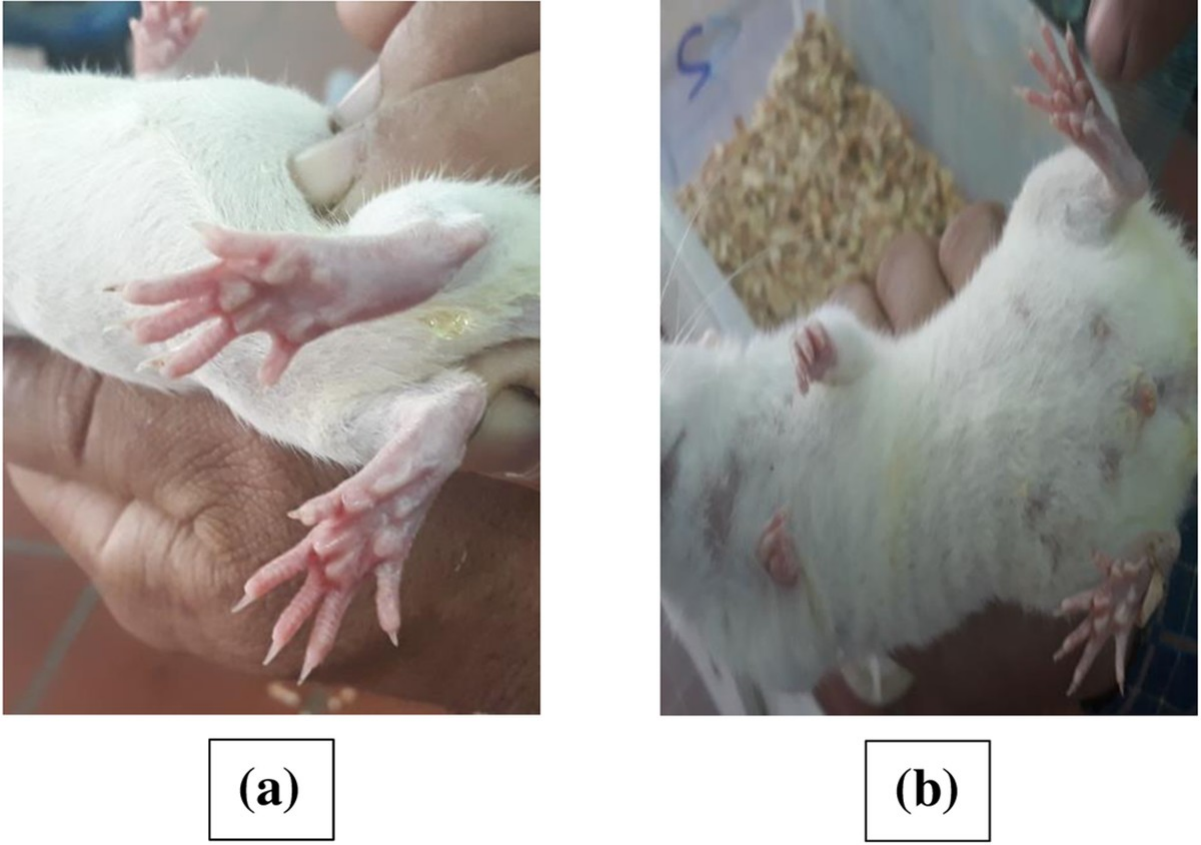
Images of right hind paw of rats showing edema before (a) and 0.5 hr after administration of optimized DCN-ODT (b).

“Fig 6A and 6B” show images of rat hind paw before oral administration of the optimized formulation and 0.5 hr after its administration, respectively, displaying significant edema inhibition. These results correlate well with the disintegration and wetting times of the optimized formulation which confirms the enhanced drug dissolution and rapid absorption of DCN.

## 4. Conclusion

In this study, ODTs loaded with optimized DCN-SDs were successfully prepared by the direct compression method and *in*-*vitro* evaluated. I-Optimal mixture experimental design was employed in order to obtain the optimized DCN-ODT formulation. Optimized formulation is composed of 250 mg solid dispersion of DCN (DCN:PEG 8000 in a ratio 1:4 w/w), 134.6 mg Pharmaburst^®^ 500 and 115.4 mg F-melt^®^. Optimized DCN-ODT gave significantly higher dissolution compared to the commercially available Diacerein^®^ capsules (*f*2 = 37.39). The *in*-*vivo* anti-inflammatory activity using the rat paw edema model confirmed the significant inhibition of edema at 0.5 hr compared to market formulation (p = 0.0465). This is explained by the enhanced solubilization and dissolution of DCN within the optimized formulation. These results suggest that formation of solid dispersions incorporable into ODTs using co-processed excipients could be promising for enhanced dissolution and rapid absorption of APIs belonging to BCS class II, through the oral mucosa. The optimized formulation could be a successful alternative for the conventional oral Diacerein^®^ capsules.

## Supporting information

S1 Table Physical characterization tests results of DCN-ODTs. (DOCX)

S1 Data *In*-*vitro* dissolution results for the prepared DCN-ODTs. (XLSX)

S1 File Statistical analysis—ANOVA results. (DOCX)

S2 File One-way ANOVA results (rat paw edema study). (DOCX)

S1 Graphical abstract (PPTX)

## Abbreviations

OA: Osteoarthritis
NSAIDs: Non-steroidal anti-inflammatory drugs
COX-II: Cyclo-oxygenase-II
DCN: Diacerein
ESCEO: European Society for Clinical and Economic Aspects of Osteoporosis and Osteoarthritis
IL-1β: Interleukin-1β
BCS II: Biopharmaceutics Classification System, class II
GIT: Gastrointestinal tract
ODTs: Orally disintegrating tablets
min: Minutes
s: seconds
DCN-SD: DCN solid dispersion
DCN-ODTs: DCN orally disintegrating tablets
APIs: Active pharmaceutical ingredients
PEG: Polyethylene glycol
DE: Dissolution efficiency
DE _(10 min)_ %: Dissolution efficiency % at 10 minutes
DE _(30 min)_ %: Dissolution efficiency % at 30 minutes
MDT: Mean dissolution time
DT: Disintegration time
WT: Wetting time
SSF: Simulated saliva fluid
*f*2: Similarity factor
ANOVA: Analysis of variance
REC: Research Ethics Committee
MCC: Microcrystalline cellulose
