# Supplementary material for: Development of orally disintegrating tablets containing solid dispersion of a poorly soluble drug for enhanced dissolution: In-vitro optimization/in-vivo evaluation
Source: PLoS One. 2020 Dec 31;15(12):e0244646. doi: 10.1371/journal.pone.0244646 (PMC7774920; doi:10.1371/journal.pone.0244646)
Supplement: S1 Table — (DOCX) [file pone.0244646.s001.docx]

**S1 Table. Physical characterization tests results of DCN-ODTs**

| Runs | Friablility (%) | Hardness (Kg) | Thickness (mm) | Drug content (%) | Weight (mg) |
| --- | --- | --- | --- | --- | --- |
| F1 | 0.88 | 5.2 ± 0.3 | 3.24 ± 0.01 | 98.20 ± 2.30 | 500.34 ± 1.24 |
| F2 | 0.82 | 5.5 ± 0.2 | 3.24 ± 0.02 | 99.70 ± 1.70 | 499.50 ± 1.83 |
| F3 | 0.89 | 4.9 ± 0.2 | 3.23 ± 0.04 | 98.12 ± 3.60 | 500.07 ± 1.54 |
| F4 | 0.85 | 5.1 ± 0.5 | 3.25 ± 0.01 | 96.76 ± 3.91 | 498.67 ± 2.40 |
| F5 | 0.92 | 5.1 ± 0.1 | 3.24 ± 0.03 | 99.63 ± 1.53 | 501.78 ± 1.12 |
| F6 | 0.87 | 5.6 ± 0.2 | 3.24 ± 0.04 | 97.82 ± 3.40 | 502.47 ± 1.42 |
| F7 | 0.83 | 5.3 ± 0.3 | 3.24 ± 0.03 | 98.46 ± 4.61 | 497.82 ± 3.41 |
| F8 | 0.79 | 5.8 ± 0.2 | 3.26 ± 0.01 | 97.64 ± 1.60 | 500.09 ± 1.71 |
| F9 | 0.93 | 4.9 ± 0.3 | 3.25 ± 0.04 | 98.42 ± 1.25 | 500.91 ± 2.42 |
| F10 | 0.98 | 4.9 ± 0.1 | 3.24 ± 0.07 | 97.47 ± 2.74 | 499.37 ± 2.71 |
| F11 | 0.75 | 5.6 ± 0.4 | 3.25 ± 0.07 | 99.63 ± 1.12 | 499.43 ± 3.63 |
| F12 | 0.69 | 5.5 ± 0.3 | 3.24 ± 0.04 | 101.23 ± 0.78 | 502.79 ± 1.21 |
| F13 | 0.89 | 5.3 ± 0.6 | 3.24 ± 0.03 | 100.78 ± 1.32 | 498.57 ± 2.46 |
| F14 | 0.87 | 5.6 ± 0.3 | 3.24 ± 0.04 | 98.23 ± 0.23 | 501.47 ± 2.14 |
| F15 | 0.78 | 5.2 ± 0.4 | 3.27 ± 0.06 | 96.19 ± 2.34 | 496.10 ± 2.69 |
| F16 | 0.74 | 5.3 ± 0.1 | 3.27 ± 0.05 | 97.67 ± 3.10 | 494.36 ± 3.74 |
