# Supplementary material for: Development of orally disintegrating tablets containing solid dispersion of a poorly soluble drug for enhanced dissolution: In-vitro optimization/in-vivo evaluation
Source: PLoS One. 2020 Dec 31;15(12):e0244646. doi: 10.1371/journal.pone.0244646 (PMC7774920; doi:10.1371/journal.pone.0244646)
Supplement: S1 File — (DOCX) [file pone.0244646.s003.docx]

1. **Statistical analysis for DE _(10 min)_ % response (Y_1_):**

**Table (1): Sequential model sum of squares for Y_1_.**

| **Source** | **Sum of squares** | **DF*** | **Mean square** | **F value** | **p-value** | **Comments** |
| --- | --- | --- | --- | --- | --- | --- |
| Mean vs. Total | 42471.10 | 1 | 42471.10 |  |  |  |
| Linear vs. Mean | 274.37 | 2 | 137.18 | 1.42 | 0.2768 |  |
| Quadratic vs. Linear | 870.71 | 3 | 290.24 | 7.54 | 0.0063 |  |
| **Sp Cubic vs. Quadratic** | **379.67** | **1** | **379.67** | **637.38** | **< 0.0001** | **Suggested** |
| Cubic vs. Sp Cubic | 1.20 | 2 | 0.60 | 1.01 | 0.4126 | Aliased |
| Residual | 4.16 | 7 | 0.59 |  |  |  |
| Total | 44001.21 | 16 | 2750.08 |  |  |  |

*DF: Degrees of freedom

**Table (2): Lack of fit of different models for Y_1_.**

| **Source** | **Sum of squares** | **DF*** | **Mean square** | **F value** | **p-value** | **Comments** |
| --- | --- | --- | --- | --- | --- | --- |
| Linear | 1251.58 | 6 | 208.60 | 350.75 | < 0.0001 |  |
| Quadratic | 380.87 | 3 | 126.96 | 213.47 | < 0.0001 |  |
| **Special Cubic** | **1.20** | **2** | **0.60** | **1.01** | **0.4126** | **Suggested** |
| Cubic | 0.000 | 0 |  |  |  | Aliased |
| Pure Error | 4.16 | 7 | 0.59 |  |  |  |

*DF: Degrees of freedom

**Table (3): Model summary statistics for Y_1_.**

| **Source** | **SD*** | **R^2^** | **Adjusted R^2^** | **Prediction R^2^** | **PRESS** | **Comments** |
| --- | --- | --- | --- | --- | --- | --- |
| Linear | 9.83 | 0.1793 | 0.0531 | -0.2261 | 1876.07 |  |
| Quadratic | 6.21 | 0.7484 | 0.6225 | 0.5178 | 737.78 |  |
| **Special Cubic** | **0.77** | **0.9965** | **0.9942** | **0.9900** | **15.28** | **Suggested** |
| Cubic | 0.77 | 0.9973 | 0.9942 |  | + | Aliased |

*SD: Standard deviation

**Table (4): ANOVA table for the special cubic model for Y_1_.**

| **Source** | **Sum of squares** | **DF*** | **Mean square** | **F value** | **p-value** | **Comment** |
| --- | --- | --- | --- | --- | --- | --- |
| **Model** | **1524.75** | **6** | **254.12** | **426.62** | **< 0.0001** | **significant** |
| *Linear Mixture* | *274.37* | *2* | *137.18* | *230.30* | *< 0.0001* |  |
| *AB* | *472.61* | *1* | *472.61* | *793.41* | *< 0.0001* |  |
| *AC* | *66.62* | *1* | *66.62* | *111.84* | *< 0.0001* |  |
| *BC* | *927.77* | *1* | *927.77* | *1557.53* | *< 0.0001* |  |
| *ABC* | *379.67* | *1* | *379.67* | *637.38* | *< 0.0001* |  |
| Residual | 5.36 | 9 | 0.60 |  |  |  |
| ***Lack of Fit*** | ***1.20*** | ***2*** | ***0.60*** | ***1.01*** | ***0.4126*** | ***not significant*** |
| *Pure Error* | *4.16* | *7* | *0.59* |  |  |  |
| Cor Total | 1530.11 | 15 |  |  |  |  |

*DF: Degrees of freedom

1. **Statistical analysis for DE _(30 min)_ %** **response (Y_2_):**

**Table (1): Sequential model sum of squares for Y_2_.**

| **Source** | **Sum of squares** | **DF*** | **Mean square** | **F value** | **p-value** | **Comments** |
| --- | --- | --- | --- | --- | --- | --- |
| Mean vs. Total | 229300 | 1 | 229300 |  |  |  |
| Linear vs. Mean | 67.75 | 2 | 33.87 | 0.016 | 0.9839 |  |
| Quadratic vs. Linear | 26811.68 | 3 | 8937.23 | 441.72 | < 0.0001 |  |
| **Sp Cubic vs. Quadratic** | **191.55** | **1** | **191.55** | **159.94** | **< 0.0001** | **Suggested** |
| Cubic vs. Sp Cubic | 0.079 | 2 | 0.040 | 0.026 | 0.9746 | Aliased |
| Residual | 10.70 | 7 | 1.53 |  |  |  |
| Total | 256400 | 16 | 16024.60 |  |  |  |

*DF: Degrees of freedom

**Table (2): Lack of fit of different models for Y_2_.**

| **Source** | **Sum of squares** | **DF*** | **Mean square** | **F value** | **p-value** | **Comments** |
| --- | --- | --- | --- | --- | --- | --- |
| Linear | 27003.31 | 6 | 4500.55 | 2944.45 | < 0.0001 |  |
| Quadratic | 191.63 | 3 | 63.88 | 41.79 | < 0.0001 |  |
| **Special Cubic** | **0.079** | **2** | **0.040** | **0.026** | **0.9746** | **Suggested** |
| Cubic | 0.000 | 0 |  |  |  | Aliased |
| Pure Error | 10.70 | 7 | 1.53 |  |  |  |

*DF: Degrees of freedom

**Table (3): Model summary statistics for Y_2_.**

| **Source** | **SD*** | **R^2^** | **Adjusted R^2^** | **Prediction R^2^** | **PRESS** | **Comments** |
| --- | --- | --- | --- | --- | --- | --- |
| Linear | 45.59 | 0.0025 | -0.1510 | -0.7678 | 47874.68 |  |
| Quadratic | 4.50 | 0.9925 | 0.9888 | 0.9859 | 380.74 |  |
| **Special Cubic** | **1.09** | **0.9996** | **0.9993** | **0.9988** | **33.69** | **Suggested** |
| Cubic | 1.24 | 0.9996 | 0.9992 |  | + | Aliased |

*SD: Standard deviation

**Table (4): ANOVA table for the special cubic model for Y_2_.**

| **Source** | **Sum of squares** | **DF*** | **Mean square** | **F value** | **p-value** | **Comment** |
| --- | --- | --- | --- | --- | --- | --- |
| **Model** | **27070.98** | **6** | **4511.83** | **3767.36** | **< 0.0001** | **significant** |
| *Linear Mixture* | *67.75* | *2* | *33.87* | *28.28* | *0.0001* |  |
| *AB* | *258.26* | *1* | *258.26* | *215.64* | *< 0.0001* |  |
| *AC* | *48.58* | *1* | *48.58* | *40.56* | *0.0001* |  |
| *BC* | *379.74* | *1* | *379.74* | *317.09* | *< 0.0001* |  |
| *ABC* | *191.55* | *1* | *191.55* | *159.94* | *< 0.0001* |  |
| Residual | 10.78 | 9 | 1.20 |  |  |  |
| ***Lack of Fit*** | ***0.079*** | ***2*** | ***0.040*** | ***0.026*** | ***0.9746*** | ***not significant*** |
| *Pure Error* | *10.70* | *7* | *1.53* |  |  |  |
| Cor Total | 27081.76 | 15 |  |  |  |  |

*DF: Degrees of freedom

1. **Statistical analysis for MDT response (Y_3_):**

**Table (1): Sequential model sum of squares for Y_3_.**

| **Source** | **Sum of squares** | **DF*** | **Mean square** | **F value** | **p-value** | **Comments** |
| --- | --- | --- | --- | --- | --- | --- |
| Mean vs. Total | 641.86 | 1 | 641.86 |  |  |  |
| Linear vs. Mean | 11.09 | 2 | 5.55 | 1.54 | 0.2509 |  |
| Quadratic vs. Linear | 24.12 | 3 | 8.04 | 3.55 | 0.0557 |  |
| **Sp Cubic vs. Quadratic** | **22.54** | **1** | **22.54** | **1477.77** | **< 0.0001** | **Suggested** |
| Cubic vs. Sp Cubic | 0.0008439 | 2 | 0.0004219 | 0.022 | 0.9786 | Aliased |
| Residual | 0.14 | 7 | 0.019 |  |  |  |
| Total | 699.74 | 16 | 43.73 |  |  |  |

*DF: Degrees of freedom

**Table (2): Lack of fit of different models for Y_3_.**

| **Source** | **Sum of squares** | **DF*** | **Mean square** | **F value** | **p-value** | **Comments** |
| --- | --- | --- | --- | --- | --- | --- |
| Linear | 46.66 | 6 | 7.78 | 399.07 | < 0.0001 |  |
| Quadratic | 22.54 | 3 | 7.51 | 385.51 | < 0.0001 |  |
| **Special Cubic** | **0.0008439** | **2** | **0.0004219** | **0.022** | **0.9786** | **Suggested** |
| Cubic | 0.000 | 0 |  |  |  | Aliased |
| Pure Error | 0.14 | 7 | 0.019 |  |  |  |

*DF: Degrees of freedom

**Table (3): Model summary statistics for Y_3_.**

| **Source** | **SD*** | **R^2^** | **Adjusted R^2^** | **Prediction R^2^** | **PRESS** | **Comments** |
| --- | --- | --- | --- | --- | --- | --- |
| Linear | 1.90 | 0.1916 | 0.0673 | -0.1935 | 69.09 |  |
| Quadratic | 1.51 | 0.6083 | 0.4125 | 0.2670 | 42.43 |  |
| **Special Cubic** | **0.12** | **0.9976** | **0.9960** | **0.9920** | **0.46** | **Suggested** |
| Cubic | 0.14 | 0.9976 | 0.9950 |  | + | Aliased |

*SD: Standard deviation

**Table (4): ANOVA table for the special cubic model for Y_3_.**

| **Source** | **Sum of squares** | **DF*** | **Mean square** | **F value** | **p-value** | **Comment** |
| --- | --- | --- | --- | --- | --- | --- |
| **Model** | **57.75** | **6** | **9.62** | **631.15** | **< 0.0001** | **significant** |
| *Linear Mixture* | *11.09* | *2* | *5.55* | *363.69* | *< 0.0001* |  |
| *AB* | *26.14* | *1* | *26.14* | *1714.04* | *< 0.0001* |  |
| *AC* | *7.27* | *1* | *7.27* | *476.41* | *< 0.0001* |  |
| *BC* | *27.02* | *1* | *27.02* | *1771.70* | *< 0.0001* |  |
| *ABC* | *22.54* | *1* | *22.54* | *1477.77* | *< 0.0001* |  |
| Residual | 0.14 | 9 | 0.015 |  |  |  |
| ***Lack of Fit*** | ***0.0008439*** | ***2*** | ***0.0004219*** | ***0.022*** | ***0.9786*** | ***not significant*** |
| *Pure Error* | *0.14* | *7* | *0.019* |  |  |  |
| Cor Total | 57.89 | 15 |  |  |  |  |

*DF: Degrees of freedom

1. **Statistical analysis for DT response (Y_4_):**

**Table (1): Sequential model sum of squares for Y_4_.**

| **Source** | **Sum of squares** | **DF*** | **Mean square** | **F value** | **p-value** | **Comments** |
| --- | --- | --- | --- | --- | --- | --- |
| Mean vs. Total | 1722000 | 1 | 1722000 |  |  |  |
| Linear vs. Mean | 382200 | 2 | 191100 | 1.08 | 0.3685 |  |
| Quadratic vs. Linear | 2275000 | 3 | 758300 | 275.80 | < 0.0001 |  |
| **Sp Cubic vs. Quadratic** | **19778.30** | **1** | **19778.30** | **23.07** | **0.0010** | **Suggested** |
| Cubic vs. Sp Cubic | 1572.24 | 2 | 786.12 | 0.90 | 0.4504 | Aliased |
| Residual | 6143.12 | 7 | 877.59 |  |  |  |
| Total | 4407000 | 16 | 275400 |  |  |  |

*DF: Degrees of freedom

**Table (2): Lack of fit of different models for Y_4_.**

| **Source** | **Sum of squares** | **DF*** | **Mean square** | **F value** | **p-value** | **Comments** |
| --- | --- | --- | --- | --- | --- | --- |
| Linear | 2296000 | 6 | 382700 | 436.08 | < 0.0001 |  |
| Quadratic | 21350.54 | 3 | 7116.85 | 8.11 | 0.0112 |  |
| **Special Cubic** | **1572.24** | **2** | **786.12** | **0.90** | **0.4504** | **Suggested** |
| Cubic | 0.000 | 0 |  |  |  | Aliased |
| Pure Error | 6143.12 | 7 | 877.59 |  |  |  |

*DF: Degrees of freedom

**Table (3): Model summary statistics for Y_4_.**

| **Source** | **SD*** | **R^2^** | **Adjusted R^2^** | **Prediction R^2^** | **PRESS** | **Comments** |
| --- | --- | --- | --- | --- | --- | --- |
| Linear | 420.84 | 0.1424 | 0.0104 | -0.6536 | 4439000 |  |
| Quadratic | 52.43 | 0.9898 | 0.9846 | 0.9775 | 60483.61 |  |
| **Special Cubic** | **29.28** | **0.9971** | **0.9952** | **0.9893** | **28736.54** | **Suggested** |
| Cubic | 29.62 | 0.9977 | 0.9951 |  | + | Aliased |

*SD: Standard deviation

**Table (4): ANOVA table for the special cubic model for Y_4_.**

| **Source** | **Sum of squares** | **DF*** | **Mean square** | **F value** | **p-value** | **Comment** |
| --- | --- | --- | --- | --- | --- | --- |
| **Model** | **2677000** | **6** | **446100** | **520.42** | **< 0.0001** | **significant** |
| *Linear Mixture* | *382200* | *2* | *191100* | *222.92* | *< 0.0001* |  |
| *AB* | *55104.69* | *1* | *55104.69* | *64.28* | *< 0.0001* |  |
| *AC* | *95941.64* | *1* | *95941.64* | *111.92* | *< 0.0001* |  |
| *BC* | *602600* | *1* | *602600* | *702.94* | *< 0.0001* |  |
| *ABC* | *19778.30* | *1* | *19778.30* | *23.07* | *0.0010* |  |
| Residual | 7715.36 | 9 | 857.26 |  |  |  |
| ***Lack of Fit*** | ***1572.24*** | ***2*** | ***786.12*** | ***0.90*** | ***0.4504*** | ***not significant*** |
| *Pure Error* | *6143.12* | *7* | *877.59* |  |  |  |
| Cor Total | 2685000 | 15 |  |  |  |  |

*DF: Degrees of freedom

1. **Statistical analysis for WT response (Y_5_):**

**Table (1): Sequential model sum of squares for Y_5_.**

| **Source** | **Sum of squares** | **DF*** | **Mean square** | **F value** | **p-value** | **Comments** |
| --- | --- | --- | --- | --- | --- | --- |
| Mean vs. Total | 333800 | 1 | 333800 |  |  |  |
| Linear vs. Mean | 40665.07 | 2 | 20332.54 | 2.77 | 0.0995 |  |
| **Quadratic vs. Linear** | **95241.68** | **3** | **31747.23** | **2039.73** | **< 0.0001** | **Suggested** |
| Sp Cubic vs. Quadratic | 0.15 | 1 | 0.15 | 0.008663 | 0.9279 |  |
| Cubic vs. Sp Cubic | 41.60 | 2 | 20.80 | 1.28 | 0.3363 | Aliased |
| Residual | 113.90 | 7 | 16.27 |  |  |  |
| Total | 469900 | 16 | 29366.86 |  |  |  |

*DF: Degrees of freedom

**Table (2): Lack of fit of different models for Y_5_.**

| **Source** | **Sum of squares** | **DF*** | **Mean square** | **F value** | **p-value** | **Comments** |
| --- | --- | --- | --- | --- | --- | --- |
| Linear | 95283.43 | 6 | 15880.57 | 976.02 | < 0.0001 |  |
| **Quadratic** | **41.75** | **3** | **13.92** | **0.86** | **0.5069** | **Suggested** |
| Special Cubic | 41.60 | 2 | 20.80 | 1.28 | 0.3363 |  |
| Cubic | 0.000 | 0 |  |  |  | Aliased |
| Pure Error | 113.90 | 7 | 16.27 |  |  |  |

**Table (3): Model summary statistics for Y_5_.**

| **Source** | **SD*** | **R^2^** | **Adjusted R^2^** | **Prediction R^2^** | **PRESS** | **Comments** |
| --- | --- | --- | --- | --- | --- | --- |
| Linear | 85.66 | 0.2989 | 0.1910 | -0.3557 | 184500 |  |
| **Quadratic** | **3.95** | **0.9989** | **0.9983** | **0.9971** | **397.86** | **Suggested** |
| Special Cubic | 4.16 | 0.9989 | 0.9981 | 0.9967 | 449.07 |  |
| Cubic | 4.03 | 0.9992 | 0.9982 |  | + | Aliased |

*SD: Standard deviation

**Table (4): ANOVA table for the quadratic model for Y_5_.**

| **Source** | **Sum of squares** | **DF*** | **Mean square** | **F value** | **p-value** | **Comment** |
| --- | --- | --- | --- | --- | --- | --- |
| **Model** | **135900** | **5** | **27181.35** | **1746.38** | **< 0.0001** | **significant** |
| *Linear Mixture* | *40665.07* | *2* | *20332.54* | *1306.35* | *< 0.0001* |  |
| *AB* | *658.54* | *1* | *658.54* | *42.31* | *< 0.0001* |  |
| *AC* | *2725.41* | *1* | *2725.41* | *175.11* | *< 0.0001* |  |
| *BC* | *47048.01* | *1* | *47048.01* | *3022.80* | *< 0.0001* |  |
| Residual | 155.64 | 10 | 15.56 |  |  |  |
| ***Lack of Fit*** | ***41.75*** | ***3*** | ***13.92*** | ***0.86*** | ***0.5069*** | ***not significant*** |
| *Pure Error* | *113.90* | *7* | *16.27* |  |  |  |
| Cor Total | 136100 | 15 |  |  |  |  |

*DF: Degrees of freedom
