# Supplementary material for: Development of orally disintegrating tablets containing solid dispersion of a poorly soluble drug for enhanced dissolution: In-vitro optimization/in-vivo evaluation
Source: PLoS One. 2020 Dec 31;15(12):e0244646. doi: 10.1371/journal.pone.0244646 (PMC7774920; doi:10.1371/journal.pone.0244646)
Supplement: S2 File — (DOCX) [file pone.0244646.s004.docx]

**One-way ANOVA results - Rat paw edema study**

**Table (1): One-way ANOVA table & multiple comparisons of the results (at 0.5 and 1 hr).**

| **0.5 hr** | | | | | |
| --- | --- | --- | --- | --- | --- |
| **Tukey's multiple comparisons test** | **Mean Diff.** | **95% CI of diff.** | **Significant?** | **Summary** | **Adjusted P Value** |
| **Control vs. OD opt** | 0.04317 | 0.001642 to 0.08469 | Yes | * | 0.0388 |
| **Control vs. DCN** | 0.001167 | -0.04036 to 0.04269 | No | ns | > 0.9999 |
| **OD opt vs. DCN** | -0.0420 | -0.08353 to -0.0004749 | Yes | * | 0.0465 |
| **1 hr** | | | | | |
| **Control vs. OD opt** | 0.2310 | 0.1112 to 0.3508 | Yes | **** | < 0.0001 |
| **Control vs. DCN** | 0.07517 | -0.04468 to 0.1950 | No | ns | 0.3732 |
| **OD opt vs. DCN** | -0.1558 | -0.2757 to -0.03599 | Yes | ** | 0.0064 |

**Table (2): One-way ANOVA table & multiple comparisons of the results (at 2 and 3 hr).**

| **2 hr** | | | | | |
| --- | --- | --- | --- | --- | --- |
| **Tukey's multiple comparisons test** | **Mean Diff.** | **95% CI of diff.** | **Significant?** | **Summary** | **Adjusted P Value** |
| **Control vs. OD opt** | 0.3043 | 0.1463 to 0.4623 | Yes | **** | < 0.0001 |
| **Control vs. DCN** | 0.1610 | 0.002992 to 0.3190 | Yes | * | 0.0443 |
| **OD opt vs. DCN** | -0.1433 | -0.3013 to 0.01467 | No | ns | 0.0887 |
| **3 hr** | | | | | |
| **Control vs. OD opt** | 0.5010 | 0.3375 to 0.6645 | Yes | **** | < 0.0001 |
| **Control vs. DCN** | 0.3452 | 0.1817 to 0.5087 | Yes | **** | < 0.0001 |
| **OD opt vs. DCN** | -0.1558 | -0.3193 to 0.007666 | No | ns | 0.0671 |

**Table (3): One-way ANOVA table & multiple comparisons of the results (at 4 hr).**

| **4 hr** | | | | | |
| --- | --- | --- | --- | --- | --- |
| **Tukey's multiple comparisons test** | **Mean Diff.** | **95% CI of diff.** | **Significant?** | **Summary** | **Adjusted P Value** |
| **Control vs. OD opt** | 0.8780 | 0.6908 to 1.065 | Yes | **** | < 0.0001 |
| **Control vs. DCN** | 0.7218 | 0.5347 to 0.9090 | Yes | **** | < 0.0001 |
| **OD opt vs. DCN** | -0.1562 | -0.3433 to 0.03101 | No | ns | 0.1350 |
