# Supplementary material for: Development of orally disintegrating tablets containing solid dispersion of a poorly soluble drug for enhanced dissolution: In-vitro optimization/in-vivo evaluation
Source: PLoS One. 2020 Dec 31;15(12):e0244646. doi: 10.1371/journal.pone.0244646 (PMC7774920; doi:10.1371/journal.pone.0244646)
Supplement: S1 Graphical abstract — (PPTX) [file pone.0244646.s005.pptx]

## Slide 1
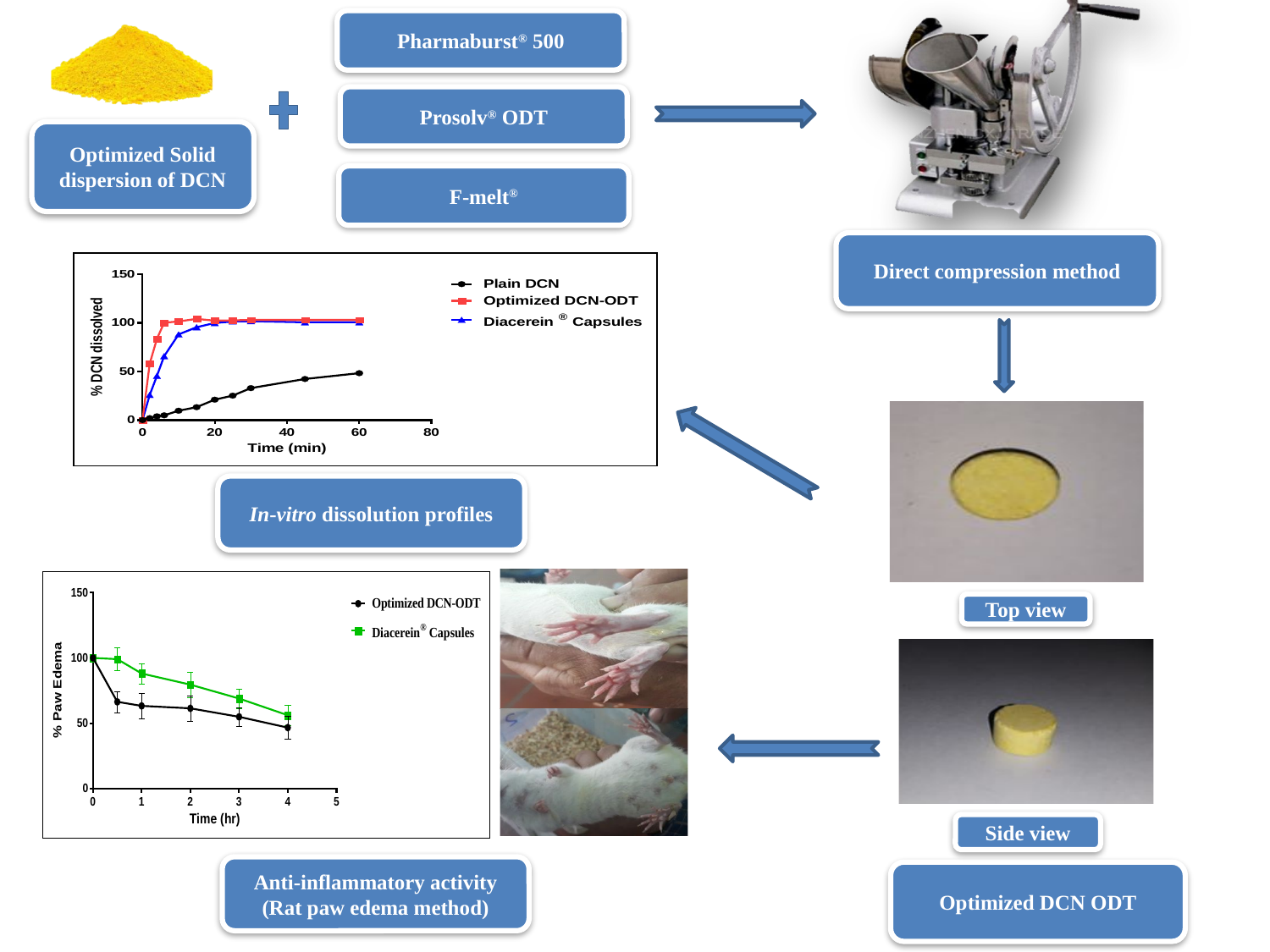

Pharmaburst® 500
Prosolv® ODT
Optimized Solid dispersion of DCN
F-melt®
Direct compression method
In-vitro dissolution profiles
Top view
Side view
Anti-inflammatory activity (Rat paw edema method)
Optimized DCN ODT
